# Economic Analysis of an Integrated Annatto Seeds-Sugarcane Biorefinery Using Supercritical CO_2_ Extraction as a First Step

**DOI:** 10.3390/ma9060494

**Published:** 2016-06-21

**Authors:** Juliana Q. Albarelli, Diego T. Santos, María José Cocero, M. Angela A. Meireles

**Affiliations:** 1 LASEFI/DEA/FEA (School of Food Engineering)/UNICAMP (University of Campinas) Cidade Universitária “Zeferino Vaz”, Rua Monteiro Lobato, 80, Campinas 13083-862, Brazil; jualbarelli@gmail.com (J.Q.A.); diego_tresinari@yahoo.com.br (D.T.S.); 2 High Pressure Processes Group, Department of Chemical Engineering and Environmental Technology, University of Valladolid, Doctor Mergelina s/n, Valladolid 47005, Spain; mjcocero@iq.uva.es

**Keywords:** integrated biorefineries, biomass valorization, supercritical fluid extraction, process modeling and simulation, *Bixa orellana* L.

## Abstract

Recently, supercritical fluid extraction (SFE) has been indicated to be utilized as part of a biorefinery, rather than as a stand-alone technology, since besides extracting added value compounds selectively it has been shown to have a positive effect on the downstream processing of biomass. To this extent, this work evaluates economically the encouraging experimental results regarding the use of SFE during annatto seeds valorization. Additionally, other features were discussed such as the benefits of enhancing the bioactive compounds concentration through physical processes and of integrating the proposed annatto seeds biorefinery to a hypothetical sugarcane biorefinery, which produces its essential inputs, e.g., CO_2_, ethanol, heat and electricity. For this, first, different configurations were modeled and simulated using the commercial simulator Aspen Plus^®^ to determine the mass and energy balances. Next, each configuration was economically assessed using MATLAB. SFE proved to be decisive to the economic feasibility of the proposed annatto seeds-sugarcane biorefinery concept. SFE pretreatment associated with sequential fine particles separation process enabled higher bixin-rich extract production using low-pressure solvent extraction method employing ethanol, meanwhile tocotrienols-rich extract is obtained as a first product. Nevertheless, the economic evaluation showed that increasing tocotrienols-rich extract production has a more pronounced positive impact on the economic viability of the concept.

## 1. Introduction

Nowadays, the image of a sustainable society is focused on a society that has a decentralized local-scale production based on local characteristics of the environment so that chemicals and energy flows can be supplied from diverse biomasses and other renewable resources. In this context, the biorefinery concept supports a “green” production platform in which different processes are integrated to convert biomass to products such as fuels, chemicals and power. It valorizes the biomass source by using its full potential, minimizing wastes and also diversifying the product matrix [1].

Supercritical fluid extraction (SFE) has demonstrated to be an ideal clean technology to be used as part of a holistic biorefinery for the recovery of bioactive compounds from plants and other vegetal materials. New perspectives on how biomasses can be better valorized with the aid of SFE process have culminated in several developments aiming full use of biomass prior to its chemical conversion, indicating that SFE can be effectively used during the first-step in biorefinery concepts [2].

Recently, many attempts were carried out to better valorize annatto seeds (*Bixa orellana* L.). Annatto seeds are a natural colorant very commonly used that imparts colors ranging from yellow to red due to the concentration of carotenoid compounds. Some strategies have been investigated by our research group (LASEFI/DEA/FEA/UNICAMP) for obtaining annatto seeds products using the combined SFE and subsequent bixin extraction aiming at higher yields with concomitant minimal carotenoid degradation. First, Albuquerque and Meireles [3] demonstrated the feasibility of using pure supercritical CO_2_ for obtaining the lipid-rich fraction of annatto seeds prior to the extraction of bixin with a more suitable extracting solvent. Secondly, Rodrigues *et al.* [4] showed that the pretreatment of the seeds with SFE had demonstrated to improve subsequent bixin extraction significantly as this pretreatment removes selectively the lipid fraction in the seeds since the solubility of bixin in pure supercritical CO_2_ is low. An additional benefit is that in this pretreatment the lipid fraction extracted is rich in tocotrienols. Tocotrienols are classified as vitamin E, they are effective antioxidant and anticancer compounds, and annatto seed oil is recognized as the largest natural source of tocotrienols [5]. Afterwards, Moraes *et al.* [6] demonstrated experimentally the viability to run an annatto seeds biorefinery concept obtaining tocotrienols-rich oil via pseudo continuous SFE prior bixin-rich extract by low-pressure solvent extraction (LPSE) with ethanol using the defatted seeds from step one. Finally, Alcázar-Alay [7] obtained promising results regarding the insertion of a fine particles separation based on milling and sieving the seeds after SFE process in order to obtain an enhanced bixin-rich biomass to be extracted by LPSE with ethanol.

Thus, the SFE process as pretreatment for the subsequent bixin extraction combined with a fine particles separation step enables the extraction of two different products from annatto seeds and one residual biomass material that could also be considered a byproduct to power production, characterizing a promising biorefinery concept for annatto seeds processing that should be evaluated in deep. The next step to consolidate this process concept is to perform studies such as economic feasibility. In this context, this paper aims at contributing to elucidate the comparative advantages of the reported experimental evaluations until now, indicating bottlenecks and essential opportunities.

The benefits of constructing a SFE plant in close proximity to an alcoholic fermentation facility that produces high purity CO_2_ as a by-product and ethanol, was already demonstrated [8]. Therefore, in the present study it was considered that the proposed annatto seeds biorefinery is located in the same site of a theoretical sugarcane biorefinery producing first and second generation ethanol. Considered these two biorefineries integrated can provide a win-win situation since the power generation facility can be shared and ethanol, CO_2_, heat and electricity can be bought at the manufacturing cost price. In this extend, this work discusses economic and energetic aspects of the proposed conceptual process design that uses SFE as an inevitable first-step in an integrated annatto seeds-sugarcane biorefinery.

## 2. Materials and Methods

### 2.1. Proposed Conceptual Process Design Description

**Figure 1:**
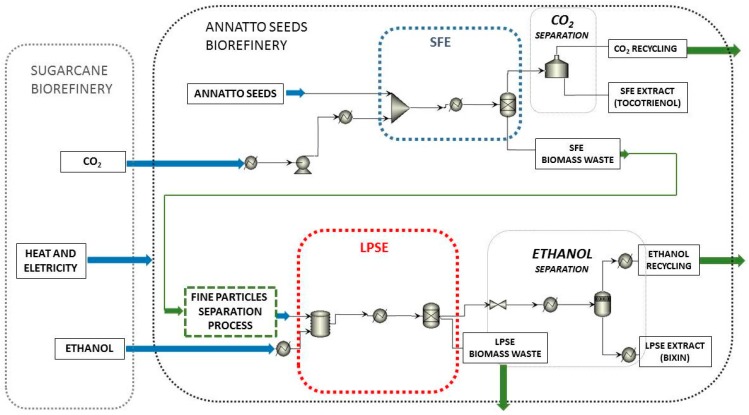
Simplified process representation for the proposed conceptual process design that uses supercritical fluid extraction process during the first stage of annatto seeds valorization processing.

The process scheme was conceptually developed based on our previously laboratory experiments. The extraction of bioactive compounds from annatto seeds in the proposed biorefinery concept was evaluated using simulation tools following the simplified process representation (Figure 1). It was investigated the supercritical fluid extraction (SFE) process as a pretreatment step to obtain tocotrienols-rich extract from the evaluated biomass and a sequential low-pressure solvent extraction (LPSE) process using ethanol as solvent to obtain bixin-rich extract. Five different process configurations were evaluated. The configurations I and II considered the SFE and the LPSE process using experimental data from Moraes *et al.* [6] and Rodrigues *et al.* [4], respectively, to calibrate the model. The configuration III considered the same process design employed in I and II scenarios, but, also prior milling and sieving of SFE residue in order to obtain only the fine particles before LPSE process, based on Alcázar-Alay [7]. Finally, the configuration IV considered only the one-step LPSE process for bixin-rich extract production based on Rodrigues *et al.* [4]. A detailed description of the considered processes is given in the following and the main parameters considered for simulation of each configuration analyzed is shown in Table 1. It was considered that the proposed annatto seeds biorefinery is located in the same site of a theoretical sugarcane biorefinery producing first and second generation ethanol, which would provide CO_2_, ethanol, heat and electricity to the annatto seeds valorization processing (Figure 1).

For all configurations, it was considered that the seeds were sent to SFE extraction without any prior treatment. As the tocotrienols-rich oils as well as bixin are majoritarily located on particle surface, any pretreatment of annatto seeds results in decreased efficiency of extraction [9]. The annatto seeds inlet flow considered in the process evaluation had chemical composition of 4.9% bixin, 12.3% moisture, 6.2% ash, 3.7 lipids, 12.1% protein and 65.7% carbohydrate [3].

#### 2.1.1. Supercritical Fluid Extraction Step

In the SFE step, CO_2_ sent to the process is initially cooled to 247 K and compressed to 20 MPa. It is then heated to the extraction temperature, 313 K, reaching the supercritical conditions. Later, the extraction vessel of 0.5 m^3^ is packed with the vegetable biomass and the supercritical fluid is passed through it. As the process was studied in a stationary regimen, it was considered multiple SFE unities working in parallel to achieve a continuous inlet and outlet material flow. For the analysis it was considered 2 supercritical extractors operating in parallel, the number of extractors was calculated considering the loading and static extraction time (5 min and 25 min, respectively), the extraction time and the depressurization and unloading time (25 min and 5 min, respectively) [6]. After the extraction process, the tocotrienols-rich extract diluted in supercritical CO_2_ is sent to a depressurization tank to separation. At this stage, the pressure is reduced to 5 MPa and temperature is set at 298 K, gasifying the carbon dioxide and separating it to be recycled to the process.

#### 2.1.2. Low-Pressure Solvent Extraction Step

The exhausted vegetable biomass matrix is removed from the extractor and sent to the LPSE process. In the LPSE process, preheated ethanol was sent into the extraction cell, maintained at the process extraction temperature, 313 or 333 K, under ambient pressure (0.1 MPa). After extraction, the remaining biomass is separated from the extraction medium by a centrifuge. This remaining biomass was considered as a biomass by-product that could be sold as biomass fuel to a power generation system. The used extracting solvent was separated from the extracted compounds by evaporation and recycled to the process.

A variation of the LPSE process was considered. Alcázar-Alay [7] recognized that a physical treatment of the SFE exhausted biomass could enable concentration of bixin prior to the LPSE process. In this process, investigated in the configuration III (Table 1), the exhausted biomass, after SFE process, was milled and sieved being only the fine fraction (23% of the total inlet mass) used for LPSE process. In this process, after fine particles separation, the exhausted biomass presented diameters varying from 300 to 1000 µm. The fine fraction comprised the fraction with diameters smaller than 300 µm, it represented 23% of the total mass inlet and 69% of the total bixin content. The considered composition of the fine fraction after separation was moisture 6%, lipids 4%, bixin 8%, total phenolic compounds 3% and others 79% [7].

The configuration IV considered the conventional one-step process for bixin extraction considered only the LPSE process as described previously, without any biomass pretreatment, being the experimental data obtained from [4].

#### 2.1.3. Sugarcane Biorefinery Integration

In the present study it was considered that the annatto seeds biorefinery is located in the same site of a sugarcane biorefinery, being the ethanol, CO_2_, heat and electricity can be bought at the manufacturing cost price. The sugarcane biorefinery here considered employed the conventional ethanol production the technologies available in modern ethanol distilleries in Brazil and also produced second generation ethanol though enzymatic hydrolysis as described elsewhere [1,8,10].

In the process modeling and simulation, it was considered that the annatto seeds biorefinery shared the cogeneration system with the sugarcane biorefinery. The cogeneration system was developed considering a steam cycle operating in a pressure of 9 MPa with extracting and condensing turbines. It was considered the burning of the residues in the analysis. The residues considered were sugarcane bagasse, leaves and biomass waste of the ethanol production process to supply the ethanol production process and the biomass waste of the annatto extraction process to supply the extraction processes; when external energy was needed to the extraction process sugarcane leaves were used. It was considered the burner as a stoichiometric reactor where air in excess (35%) was used to the reaction. The off-gases were cooled to 473 K and the heat was used as the heat source to the steam network [1,8,10].

### 2.2. Process Modeling and Simulation Description

A flowsheet model of the integrated annatto seeds-sugarcane biorefinery was developed using the commercial software Aspen Plus^®^ and the process integration and the thermo-economic evaluation was carried out using the platform OSMOSE. The thermodynamic model used to represent the process was RK-ASPEN model when supercritical fluid extraction was considered and UNIQUAC model for low pressure processes. For more details about the modeling and simulation performed in Aspen Plus^®^ v 8.4 (AspenTech, Bedford, MA, USA) see Appendix A. OSMOSE simulation tool was used in its basic level to perform thermal integration and economic analysis. OSMOSE (OptimiSation Multi-Objectifs de Systemes Energetiques integres, which means “Multi-Objective OptimiZation of integrated Energy Systems”) is a computation platform that was built in MATLAB (MATrix LABoratory, MathWorks, Natick, MA, USA), developed and continuously improved at École Polytechnique Fédérale de Lausanne in Switzerland for the design and analysis of integrated energy systems. The platform allows one to link Aspen Plus^®^ software for a complete suite of computation and result analysis tools. This platform relies on the methodology described in detail in Appendix B. In this study, the problem resolution was carried out following the steps:

1. Process data is gathered through literature search
2. Aspen Plus^®^ flowsheeting software was used to model mass and energy flows of the process. The model was used to calculate the associated heat and power balances
3. Pinch analysis methodology [11] was used to perform the thermal integration of the process aiming at the reduction of process steam requirements
4. An economic model was developed in the OSMOSE platform using data obtained from the flowsheeting software Aspen Plus^®^ and the results obtained by the thermal integration model (for more details see Appendix A and Appendix B).

The proposed biorefinering configurations were evaluated regarding productivity and economic process indicators.

#### 2.2.1. Process Productivity Indicators

From the data obtained in the simulation it was possible to determine the tocotrienols and/or bixin productivity of the evaluated process configuration, which represents the total amount of bioactive compounds produced annually.

#### 2.2.2. Economic Indicators

In order to accomplish an economic evaluation of the process viability at industrial scale, lab results were scaled-up considering that the same performance would be obtained. This criterion, which has been used by other authors for SFE processes [12,13], assumes that the process will have identical performance with respect to yield at the laboratory and industrial scales if the same process conditions are used (temperature, pressure, extraction time, bed density, Solvent mass to Feed mass ratio (S/F), *etc.*). To calculate the total investment cost, the major process equipments were roughly sized and their purchase cost were calculated and adjusted to account for specific process pressures and materials using correlations from literature [14,15]. The total investment cost was then calculated using multiplication factors to take into account indirect expenses like installation costs, contingencies and auxiliary facilities. All costs had been updated by using the Marshall and Swift Index.

Cost of manufacturing (COM) estimation for the proposed biorefinery concept was accomplished was based on the methodology of Turton *et al.* [12], in which variable cost (VC) (operational costs which are dependent on the production rate and consist in raw material costs, operational labor, utilities, among others), fixed costs (FC) (do not dependent on production rate and include territorial taxes, insurance, depreciation, *etc.*) and general expenses (GE) (cover business maintenance and include management, administrative sales, research and development costs) are calculated. These three components are estimated in terms of five main costs: fixed capital investment (FCI), cost of utilities (CUT), cost of operational labor (COL), cost of waste treatment (CWT) and cost of raw materials (CRM). The raw material cost is mainly related to the vegetable biomass and the extracting solvent lost during the process. Utility costs considered the electricity and the cooling requirements under 293 K. It was considered that the necessary steam, after thermal integration, was supplied by the cogeneration system by the burning of the residue and therefore presented no cost. The cost of waste treatment may be neglected as the residue of the process was considered to be used as fuel to the cogeneration system and the reactants CO_2_ and ethanol are recycled to the process. The COM was calculated as presented in Equation (1).

COM = (VC + FC + GE) × (1 + 0.03COM + 0.11COM + 0.05COM)
(1)

In which 0.03COM represents the royalties; 0.11COM the distribution and selling and 0.05COM the research and development investments.

The profitability ratios selected in this study to evaluate the economic feasibility of the scenarios are presented from Equation (2) to Equation (10), which lead to a true disclosure regarding the economy of the proposed biorefinery concept. The revenue is calculated considering the sale of the products tocotrienols-rich extract and bixin-rich extract in a year, and the depreciation considered was 1% of the total investment.

(2) $$\text{Annualized investment}=\text{Total Investment Cost}\times \frac{i\times {\left(i+1\right)}^{n}}{{\left(i+1\right)}^{n}-1}$$

Annual benefit = revenue − COM − annualized investment
(3)

Gross profit = revenue − COM,
(4)

Expenses = COM + depreciation,
(5)

Net profit = (revenue − expenses) × (1 − tax rate)
(6)

Cash-flow = net profit + depreciation
(7)

Gross margin = gross profit/annual revenue
(8)

Return on investment (ROI) = annual net profit/total capital investment
(9)

(10) Payback time = total capital investment/annual net profit

Table 2 shows the list of assumptions that support the economic assessment results.

#### 2.2.3. Sensitivity Analysis

A sensitivity analysis was accomplished in order to forecast the influence of the optimization of the SFE process over the overall biorefinery for the best biorefinery configuration studied. Data from [6] of tocotrienol extraction over different S/F ratios was used to simulate the SFE. As the impact of the different SFE extraction conditions over the LPSE parameters was not studied experimentally, an approximation for the bixin extraction yield was given by the Equation (11). Most probably, the effect of SFE condition on bixin yield will not be linear as supposed, but only with experimental studies it will be possible to extract a reliable equation. For now, to enable an evaluation of the biorefinery as it is it was considered that the calculated BY could vary ±15%.

(11) $$BY={BY}_{R}-\left({BY}_{R}-{BY}_{0}\right)\left(\frac{t_{R}-t}{t_{R}}\right)$$

*BY*_R_ is the bixin yield obtains experimentally for the configuration studied [7]; *BY*_0_ is the bixin yield obtains experimentally when SFE is not considered [4]; *t*_R_ is the SFE treatment time for obtaining the *BY*_R_; t is the evaluated SFE treatment time.

## 3. Results and Discussion

### 3.1. Annatto Seeds-Sugarcane Biorefinery Evaluation: Process Productivity Indicators

**Figure 2:**
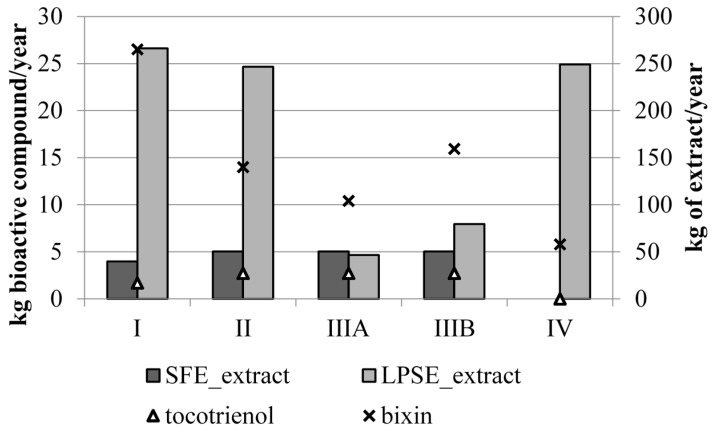
Productivity of SFE extract, LPSE extract, tocotrienols and bixin for the evaluated annatto seeds-sugarcane biorefinery configurations.

Figure 2 shows the productivity of SFE extract, LPSE extract, tocotrienols and bixin for the evaluated annatto seeds-sugarcane biorefinery configurations. The SFE process in the proposed biorefinery enabled product diversification by producing not only bixin but also tocotrienols–rich extract. It should be noted that currently, the bioactive compound bixin is the unique target extracted compound by the food industries, which has being obtained mostly by the extraction with an alkaline solution [16].

The productivity for tocotrienols is around 4 to 16 times lower than the bixin production, depending on the evaluated configuration. Tocotrienols productivity for configurations II and III were the same as the same SFE process parameters, (S/F of 3.5), was considered.

Configuration I presented the highest bixin productivity mainly due to the high bixin extraction presented by Moraes *et al.* [6]. In their study it was possible to extract 3.4 ± 0.3 g of bixin/100 g of annatto seeds in the sequential SFE-LPSE process, but no information on the initial amount of bixin is given. Therefore, considering the maximum average content of bixin would be 6.3 g of bixin/100 g of annatto seeds [3], the bixin recovery yield would be of 53.97%, quite superior than the other bixin recovery yield analyzed for LPSE (Table 1). For Configuration IIIB (S/F of 20), it is possible to notice that the seeds milling, sieving and separation process step has a positive impact on the bixin extraction, enabling a higher bixin recovery yield through LPSE process (Table 1) and a higher bixin productivity (kg bioactive compound/year) (Figure 2). In this configuration, the fine particle separation process step acts as bixin concentrator. The use of a specific biomass fraction after milling and separation with a particular characteristic can also be observed for other biomasses [17]. The milling process and sequential separation of the diameters by sieving can allow the separation of the different plant structures. This gives different characteristics to each biomass fraction obtained after sieving, as each plant structure has its function on the vegetal matrix and therefore different characteristic can be found. In the present study, it is possible that after milling and separation of the fine fraction was responsible to concentrate the vegetal structure responsible for holding bixin. According to Silva *et al.* [17] better results for cellulose saccharification when using only fine fraction of sugarcane bagasse comparing with the use of the complete bagasse to second generation process. Improved yields of hydrolysis was obtained by separating the bagasse in fiber and pith, and using only the pith fraction (fine particles), composed by parenchyma cells, for second generation ethanol production. These cells demonstrated to have lower lignin content comparing to the bagasse fibers, which facilitated the enzymatic attack to cellulose requiring a mild pretreatment step with no catalyst consumption. Thus, an in deep vegetal structure analysis should be further done in order to better understand the phenomenon involved in the annatto seeds fine particles separation.

Configuration IV resulted in the highest LPSE extraction yield but with the lowest bixin recovery. When a previous SFE process is considered, it was possible to increase bixin production from 1.8 to 4.6 times and also promote higher bixin recovery yield (Table 1). This improvement on bixin extraction was explained by Rodrigues *et al.* [4] being associated to the removal of lipids together with the tocotrienols-rich fraction. Vatai *et al.* [18] performed extractions of phenolic compounds from elder berry and grape marc also in two stages, combining SFE and conventional extractions with organic solvent, observing also that pretreatment with supercritical carbon dioxide improved the recovery process by removing the non-polar components becoming the polar polyphenols more accessible.

Table 3 presents the consumption of resources and the biomass waste generated by the configurations evaluated. In the SFE process, configurations II and III considered a higher S/F than Configuration I (Table 1), what leads to a higher CO_2_ consumption. Though CO_2_ consumption is higher, a lower CO_2_ consumption ratio (kg/t tocotrienols) is presented as tocotrienols production is also higher. Similar behavior is found for solvent consumption ethanol in the LPSE process, in which for configurations IIIA and IIIB the higher bixin productivity enabled a low ethanol consumption rate. The electricity consumption increased significantly for configurations IIIA and IIIB due to the introduction of the milling-sieving step. Energy consumed in fine particles separations step corresponded to 73% of the overall electricity consumption for the configurations IIIA and IIIB.

Configuration produced the highest biomass waste amount in relation to the amount of bioactive compound extracted, as the biomass was submitted to only one extraction process. Configurations IIIA and IIIB also presented high biomass waste produced as only 23% of total amount of first step biomass residue was considered for LPSE extraction. The biomass residue formed by annatto seeds is extremely rich in carbohydrates [19]. In the present study, it was considered as fuel for the cogeneration system to close the heat balance in the process but it could be considered as a by-product with higher added-value and other biomass waste of the annatto seed production chain, *i.e.*, leaves and agricultural waste, could be used as fuel. It should be noted that there is also a considerable residual bixin in the waste that should be addressed and valorized. In configuration III, the separation of the fine fraction to LPSE enabled the separation of the biomass waste in a bixin-rich waste after extraction and a carbohydrates-rich waste that is not used for extraction. This could allow the valorization of the two different streams in different and more truthful way. The bixin-rich waste could be sold as a second grade colorant while the carbohydrate fraction could be used for hydrolysis or chemical modification. The sugar oligomers and monomers produced by the hydrolysis can be included in industrial processes for food, chemicals and sustainable fuel production [20]. Likewise, it could be considered the starch modification to development of specific properties such as solubility, texture, adhesion and tolerance in this new product [21].

### 3.2. Annatto Seeds-Sugarcane Biorefinery Evaluation: Economic Indicators

**Figure 3:**
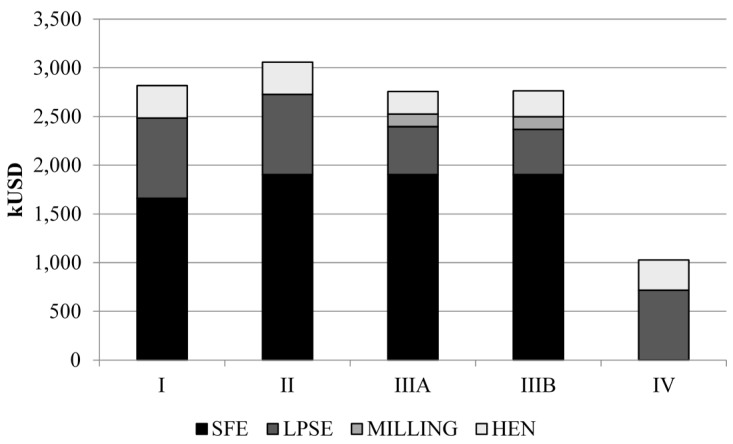
Investment cost for the evaluated annatto seeds-sugarcane biorefinery configurations.

Regarding the economic parameters evaluated, similar investment cost is found for configurations I, II and III (Figure 3). The SFE process presented the higher share in the investment, from 59% to 69% of the investment depending on the configuration. The lower SFE cost is found for configuration I. Although the SFE extractor size adopted for all configurations is the same, 0.5 m^3^, the S/F considered for configuration I is smaller, therefore, requiring smaller solvent pumps and recirculation equipment.

LPSE investment is lower for Configuration III. In this configuration, the amount of ethanol used in the LPSE is higher, S/F of 10 and 20, than configurations I and II, S/F 8. However, the impact of volume due to higher solvent feed ratio is minimized as a smaller amount of biomass is sent to the process, 23%, ending up requiring smaller extractions than other configurations. The LPSE extractor investment in configuration III is around 54% less than configurations I and II as less vegetable biomass is sent to the process. The fine particles separations unity, that comprises the mill and the sieve separator, corresponded to only around 4.7% of the investment for configuration III. The use of the fine fraction with concentrated bixin content (Configuration III) proved to be strategic for decreasing the overall investment cost even considering the cost of the fine particles separations unity (Figure 3).

The cost of manufacturing (COM) calculated for the evaluated configurations was similar for all evaluated configurations (Table 4). Configuration IV presented the lowest COM (MUSD/year) due to the lower investment necessary and number of workers as SFE inclusion is not considered. The main contribution for the COM was the variable cost in which the vegetable biomass annatto seeds contributed in average with 90%, corroborating literature findings [12]. The COM calculated in terms of bioactive compound (COM bioactive) shows the mean value for tocotrienols and bixin production, by analyzing configuration IV it is possible to perceive that the SFE process contributed to decrease the bioactive compound production cost. This was beneficial for the annatto seeds valorization process as increased its overall economic attractiveness.

In order to evaluate the economic feasibility of the configurations other economic indicators were calculated. The revenue was calculated considering the sale of the products tocotrienols-rich extract and bixin-rich extract. The price for the extracts was calculated taking in to account that only the amount of bioactive compounds presented has economic value. The use of SFE process during the annatto seeds processing proved to allow the economic viability of the overall extraction process (Table 5), at the parameters of cost and product price assumed. Configuration IV, in which SFE is not considered, presented the lowest economic indicators considering investment (total investment and annualized investment) and also expenses, but, due to the low bixin recovery yield cash-flow of this configuration was negative and therefore presented no economic viability. The amount of tocotrienols extracted was a decisive factor to increase economic viability as the price for this bioactive compound is 110 times higher than the bixin price. Therefore, configuration I, that presented lower tocotrienols production but the highest bixin production offered itself as the less economically attractive configuration of combined SFE and LPSE processes studied. The economic indicators for configurations II and III were similar. On the other hand, regarding return on investment (ROI) and payback time indicators the results configuration IIIB was slightly better than configuration IIIA and II, respectively, indicating that these configurations would present the best scenario for rapid return on the invested capital but after the investment has returned configuration IIIB would give the higher profit.

Even though the optimization of the evaluated processes could bring a different economic scenario to display, the overall picture would not be modified. Supercritical pretreatment prior to bixin extraction increases 3 times the investment but it also increases bixin extraction yield [4] and produces the tocotrienol extract that has a selling price 110 times higher than bixin. With this, even with the optimization of Configuration IV, Configurations I–III would present better economic viability than Configuration IV. The process with higher impact in the economic viability of the biorefinery is the SFE as it represents around of 63% of the investment and produces the product with higher selling value. The impact of optimizing the SFE process is clear when comparing Configurations I and II. Both presents the same SFE and LPSE configuration, but for configuration II S/F in the SFE extraction is higher resulting in a tocotrienol production 10% higher. It increases the economic viability of the process, that can be seen in the increase of gross profit and decrease of payback time, even with the decrease of 60% in bixin production. Comparing configurations II and III, it is possible to produce an extract with higher bixin concentration by separating the fine fraction with similar economic viability.

A sensitivity analysis was undertaken to evaluate the effect on SFE S/F on the overall biorefinery economic indicators for configuration IIIB. The increase tocotrienol production also reflects on an increase on bixin extraction (Table 6), but with the increase of S/F 3.5 to 11 no statistically significant improvement on the bixin extraction yield was noticed experimentally [7]. Therefore S/F values higher than 4.5 were not investigated. With the increase on the SFE S/F, investment and COM slightly increases. SFE S/F of 1.8 is on the limit of presenting positive cash-flow for the process, but the economic indicatives as the payback shows that it is a not feasible configuration economically. It was possible to note that the higher the tocotrienol extraction, the higher it will be the economic benefit of the process for the evaluated SFE S/F parameters even with the increase in investment and COM.

### 3.3. Benefis of the Integration of the Annatto Seeds biorefinery to a Sugarcane Biorefinery

The different configurations for the annatto seeds biorefinery were evaluated always considering the installation of the process integrated with the sugarcane biorefinery, in which the cogeneration facility can be shared and ethanol, CO_2_, heat and electricity was send from sugarcane biorefinery to annatto seeds biorefinery. The economic benefit of placing an SFE process inside a sugarcane biorefinery was stated previously by Santos *et al.* [8], considering Brazilian ginseng roots as model vegetable biomass. Placing one biorefinery next to other can lead to shared waste and water treatment, shared utility production, use process wastes as raw-material in new processes and other benefits depending on the type of biomass and/or biorefinery studied. In this context, we find the recent studies about the synergetic effects of integrating a sugarcane-based biorefinery with a microalgae-based biorefinery [22,23].

The present study considered the waste vegetable biomass from the annatto seed biorefinery to be burned as fuel in the shared cogeneration system. For the annatto seeds biorefinery to have its own burner, the total investment would increase around 6.2% for the combined SFE-LPSE configurations and 17.1% for the one-step LPSE process, configuration IV.

The hypothetical existing facility considered here already burns 95 t/h of biomass material (sugarcane bagasse, sugarcane leaves and other biomass process wastes) producing approximately 2400 MW of heat and 64.8 MW of electricity of which 67% is not used in the process and sold to the grid. The annatto biomass waste was an interesting alternative to be burned at the cogeneration system as it presented low water content and residual ethanol from the LPSE extraction (after centrifugation of biomass less than 5% of ethanol used in the extraction remains in the waste). According to Brazilian burners manufactures, it would be possible burn biomass with different characteristics (*i.e.*, low moisture content) than sugarcane bagasse up to 15% of the burner inlet [24]. The annatto seeds biomass waste represents less than 1%.

With the burning of the annatto biomass waste it was possible to produce approximately 4000 KW (4 MW) of heat (an average of 9.5 kWh/kg of waste biomass, 4000 kW/420 kg/h) (Table 7). The heat produced by burning the waste could successfully supply the heat demand of the processes before thermal integration. The heat demand before thermal integration was around 768–780 kW for configuration I, II and IV and of 223 and 433 for configurations IIIA and IIB, respectively. Lower heat demand is found for configuration III as LPSE process not only has a smaller scale, due to lower biomass inlet, as the considered temperature that the process is performed is lower, 313 K instead of 333 K (Table 1). After thermal integration, the heat demand was reduced around 65.5%. With lower heat consumption the excess heat could be used to electricity production in the cogeneration system, the generated electricity could be used to supply the electricity demand of the process decreasing its cost. This scenario was not evaluated and could be further investigated with more accuracy when further information on the waste material could be supplied as full chemical and thermal characterization of the residue.

The cold demand above 293 K was considered to be cooled with cooling water from the sugarcane process, and therefore no cost for this cooling fluid was considered. The cooling demand under this temperature was considered to be supplied by a cooling fluid with cost of 0.028 USD/kWh (Table 2).

Another relevant aspect of the synergy between both biorefineries are the solvents used. In the annatto seed biorefinery CO_2_ and ethanol are used as solvent, both are produced at the sugarcane biorefinery (Figure 1). Therefore, it was considered in the economic analysis that the cost for CO_2_ and ethanol reposition was the production cost of these solvents in the sugarcane process. The solvents, CO_2_ and ethanol, also need rectification after some time of use as during the process they end up retaining water from the biomass. Without considering solvent recycle in a closed loop, Aspen Plus^®^ simulations indicated that the amount of water in the flow the CO_2_ increases from 0% to 0.05% and in the ethanol increases from 0.7% to 0.95%. To recover the solvents without the integration between biorefineries would induce an increase in the investment and COM for the annatto seeds biorefinery to the acquisition and operation of the rectification columns for ethanol purification and the CO_2_ purification system. One alternative would be to buy new solvents and discard the old solvents, this alternative would increase the expenses and a waste treatment process would be necessary. With the integration of these two biorefineries the used ethanol might be send to the sugarcane process for purification using the already available infrastructure.

## 4. Conclusions

The use of supercritical fluid extraction (SFE) process prior to biomass transformation can be an alternative to enhance full biomass utilization and lead to product diversification in a biorefinery concept. The economic evaluation of experimental developed strategies for annatto seeds valorization using process modeling and simulation tools proved that SFE is decisive to the economic feasibility of the biorefinery concept as its enables higher bixin-rich extract production and associated with fine particle seeds separation also promoted an extract with higher bixin concentration. The SFE process presented the higher share in the investment, from 59% to 69% of the investment depending on the configuration. Lower low-pressure solvent extraction (LPSE) investment, total investment and cost of manufacturing (COM) for the integrated SFE-LPSE process was found for the alternatives that considered prior fine particles separations before LPSE process. Due to the high concentration of the bixin in the fine fraction, this configuration requires less solvent and smaller LPSE infrastructure. Tocotrienols-rich extract production through SFE presented high positive impact on the economic indicators studied, the higher the tocotrienols production the higher the revenue, cash-flow, gross margin, ROI and lower the payback time. Negative cash-flow, representing an economically unfeasible biorefinery at the economic parameters evaluated, was found for the one-step LPSE process as only bixin extract, which resulted in lowest bixin recovery yield of the evaluated configurations. With the integration of the annatto seeds biorefinery with a sugarcane biorefinery, the cogeneration facility could be shared and also the solvents used during the annatto seeds processing could be recycled directly at its production site, *i.e.*, at the sugarcane biorefinery. This leads to a decrease in the total investment cost for implementing the annatto seeds biorefinery concept proposed. In addition, the integrated perspective leads to a reduction of the COM as the solvent recycle and utility generation steps do not have to be administered and maintained by the annatto seeds biorefinery. Thermal integration decreased significantly, around 65%, the thermal demand of the annatto seeds-sugarcane biorefinery. Waste biomass from the two-step process was used for heat production, producing an average of 9.5 kWh/kg of waste biomass, what was enough to supply the heat demand of the process. Based on the process indicators studied, the best configuration evaluated was the integrated SFE-LPSE process with the fine particles separation step before LPSE, considering the LPSE ethanol solvent ratio of 20 (Configuration IIIB).

## Figures and Tables

**Table 1:** Main parameters considered for simulation of each configuration analyzed.

Configuration	I	II	IIIA	IIIB	IV	Unit
SFE						
Extraction pressure	20	20	20	20	-	(MPa)
Extraction temperature	313	313	313	313	-	(K)
Solvent mass to Feed mass ratio (S/F)	1.8	3.5	3.5	3.5	-	
Extraction yield	1.69	2.14	2.14	2.14	-	(g of extract/100 g of annatto seeds, dry basis)
Tocotrienols content in the extract	11.33	14.44	14.44	14.44	-	(g of tocotrienols/100 g of extract)
CO_2_ Recovery pressure	5	5	5	5	-	(MPa)
CO_2_ Recovery temperature	273	273	273	273	-	(K)
Particles separations before LPSE	no	no	Yes	Yes	No	
*LPSE*						
Extraction pressure	0.1	0.1	0.1	0.1	0.1	(MPa)
Extraction temperature	333	333	313	313	333	(K)
Solvent mass to Feed mass ratio (S/F)	8	8	10	20	8	
Extraction yield	11.33	10.56	8	10	10.56	(g of extract/100 g of annatto seeds, dry basis)
Bixin recovery yield	53.97	28.49	32	49	11.81	(%, g of bixin extracted/theoretical g of bixin that can be obtained from inlet material ×100)
Ethanol recovery pressure	0.016	0.016	0.016	0.016	0.016	(MPa)
Ethanol recovery temperature	348	348	348	348	348	(K)

**Table 2:** List of assumptions of the economic analysis of the proposed annatto seed-sugarcane biorefinery.

Economic Data	Value	Unit
Project lifetime	25	(years)
Construction and startup	2	(years)
Depreciation	10	(years)
Interest rate	15	(% per year)
Days worked in a year	320	(days/year)
Raw materials prices		
Annatto seeds	2.0 ^1^	(USD/kg)
Ethanol	0.49 ^2^	(USD/L)
CO_2_	0.30 ^3^	(USD/kg)
Electricity	0.05 ^4^	(USD/kWh)
Cold demand under 293 K	0.028 ^5^	(USD/kWh)
Product prices		
Tocotrienols	1732.70 ^6^	(USD/kg bioactive compound)
Bixin	15.75 ^6^	(USD/kg bioactive compound)

^1^ data from [3]; ^2^ ethanol production cost calculated by the simulation; ^3^ data from [8]; ^4^ data from [1]; ^5^ data from [12]; ^6^ calculated based on a medium value from different annatto seed extracts found in the market.

**Table 3:** The consumption of resources and the biomass waste generated by the configurations evaluated.

Configuration	I	II	IIIA	IIIB	IV	Unit
Annatto Seeds	2688	2688	2688	2688	2688	(t/year)
CO_2_	39.08	21.06	21.06	21.06	0	(kg/t tocotrienols)
Ethanol	1.12	2.11	0.83	0.44	5.18	(L/kg bixin)
Electricity	2.01	5.56	23.39	16.40	3.22	(kWh/kg bioactive compound)
Cold demand (under 293 K)	73.56	88.66	88.66	88.66	0	(kWh/kg tocotrienols)
Biomass waste	28.33	47.95	72.83	50.51	139.42	(kg/kg bioactive compound)

**Table 4:** Cost of manufacturing (COM) calculated for the evaluated annatto seeds-sugarcane biorefinery configurations.

Configuration	I	II	IIIA	IIIB	IV	Unit
Variable cost	74.9	74.4	75.0	75.0	79.5	(%)
Fixed cost	8.3	8.7	8.1	8.2	4.0	(%)
General cost	0.9	0.9	0.9	0.9	0.5	(%)
COM	7.99	8.07	7.99	7.98	7.17	(MUSD/year)
COMextract ^1^	26.1	27.2	82.2	61.4	28.8	(USD/kg of extract)
COMbioactive ^2^	105.4	179.8	226.4	159.2	459.7	(USD/kg of bioactive compound)

^1^ COM calculated in terms of extract; ^2^ COM calculated in terms of bioactive compound.

**Table 5:** Economic indicators for the evaluated annatto seeds-sugarcane biorefinery configurations.

Economic Indicator	I	II	IIIA	IIIB	IV	Unit
Total investment	2817.31	3058.78	2756.11	2762.54	1027.33	(kUSD)
Annualized investment	310.38	336.98	303.64	304.34	113.18	(kUSD)
Annual benefit	657.17	4817.00	4785.77	5020.14	−7034.16	(kUSD)
Income products	8953.65	13,226.76	13,075.11	13,309.25	245.55	(kUSD)
Expenses	8267.83	8378.65	8261.32	8261.02	7269.27	(kUSD)
Net profit	617.24	4363.30	4332.41	4543.41	−6321.34	(kUSD)
Cash flow	898.97	4669.17	4608.02	4819.66	−6218.61	(kUSD)
Gross profit	967.55	5153.98	5089.40	5324.48	−6920.98	(kUSD)
Gross margin	0.11	0.39	0.39	0.40	-	(%)
ROI	0.22	1.43	1.57	1.64	-	(%)
Payback time	4.6	0.7	0.6	0.6	-	(years)

**Table 6:** Sensitivity analysis of SFE S/F for configuration III.

S/F		1.8		2.7		3.5 *	4.5	
Tocotrienol	(kg/t annatto)	1.7		2.2		2.7	3.2	
Bixin	(kg/t annatto)	10.3	±1.5	13.4	±2.0	15.9	17.4	±2.6
COM	(MUSD)	7.9		8.0		8.0	8.0	
Total Investment	(MUSD)	2.5		2.8		2.8	2.8	
Payback	(years)	27.1	+47; −11	1.2	±0.04	0.6	0.4	±0.01
Cash Flow	(MUSD)	0.3	±0.06	2.66	±0.08	4.82	7.01	±0.10
ROI	(%)	0.037	±0.02	0.867	±0.03	1.645	2.446	±0.04
Gross Margin	(%)	0.043	±0.01	0.268	±0.01	0.400	0.493	±0.00

* Uncertainties were not considered for this S/F point as BY correspond to the experimental value (BY_R_) obtained by [7].

**Table 7:** Heating and cooling demand and steam generated using the annatto seeds residue for the evaluated annatto seeds-sugarcane biorefinery configurations.

Configuration	I	II	IIIA	IIIB	IV	Unit
*Before thermal integration*						
Heating demand	768	770	223	433	780	(kW)
Cooling demand	−751	−749	−213	−420	−764	(kW)
*After thermal integration*						
Heating demand	269	268	78	148	266	(kW)
Cooling demand	−248	−243	−65	−135	−250	(kW)
*Heat demand not considered in the thermal integration*						
Cooling demand under 293 K	−43	−84	−84	−84	−43	(kW)
*Using the waste as fuel*						
Solid waste	422.2	422.3	412.4	374.7	428.3	(kg/h)
Moisture content in the waste	1.4	1.4	13.9	13.8	1.5	(%)
Residual ethanol in the waste	32.4	32.2	52.8	35.4	32.5	(%)
Steam generated	4000.5	4004.2	4075.7	4097.4	4013.8	(kW)

**Figure A1:**
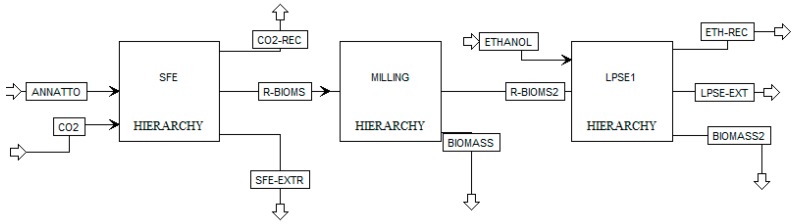
Overall annatto seeds biorefinery flowchart developed in Aspen Plus^®^.

**Figure A2:**
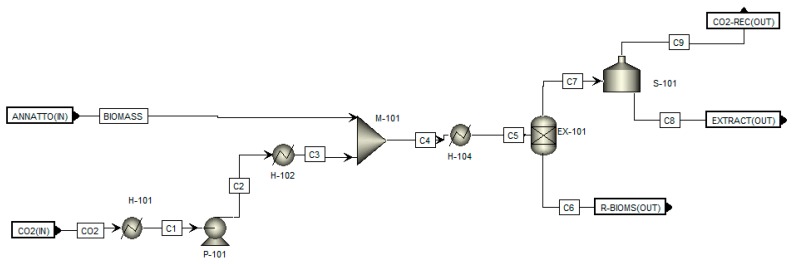
Flowchart of the supercritical fluid extraction (SFE) process for obtaining tocotrienols-rich extract from annatto seeds developed in Aspen Plus^®^.

**Figure A3:**
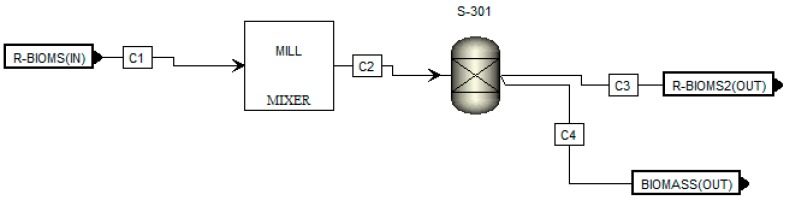
Flowchart of the fine particles separations process developed in Aspen Plus^®^.

**Figure A4:**
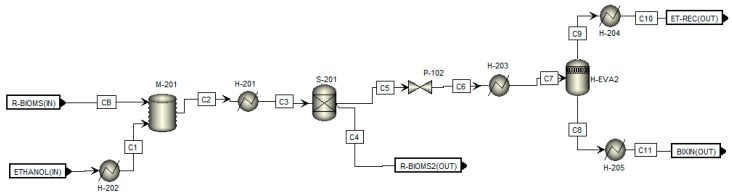
Flowchart of the low-pressure solvent extraction (LPSE) process for obtaining bixin-rich extract from annatto seeds developed in Aspen Plus^®^.

**Figure B1:**
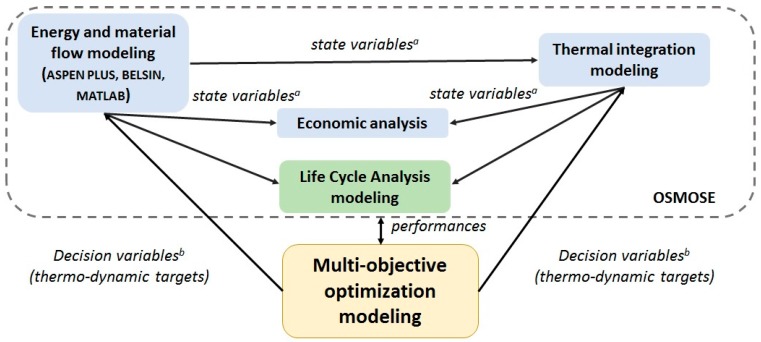
Modeling methodology possible in the OSMOSE (OptimiSation Multi-Objectifs de Systemes Energetiques integres) platform.

## Abbreviations

The following abbreviations are used in this manuscript:

SFE	Supercritical Fluid Extraction
LPSE	Low-Pressure Solvent Extraction
S/F	Solvent mass to Feed mass ratio
OSMOSE	OptimiSation Multi-Objectifs de Systemes Energetiques integres
MATLAB	MATrix LABoratory
COM	COst of Manufacturing
VC	Variable Cost
FC	Fixed Costs
GE	General Expenses
FCI	Fixed Capital Investment
CUT	Cost of Utilities
COL	Cost of Operational Labor
CWT	Cost of Waste Treatment
CRM	Cost of Raw Materials
ROI	Return On Investiment

## Appendix A

In this appendix Aspen Plus^®^ simulation details are described.

The thermodynamic model used to represent the process was RK-ASPEN model when supercritical fluid extraction was considered and UNIQUAC model for low pressure processes. The RK-ASPEN model can be applied to the SFE process as it is particularly suitable for modeling a mixture of light gases (such as CO_2_,) at medium to high pressures, with polar components. The RK-ASPEN property method distinguishes between the subcritical and supercritical components and applies either the Mathias alpha function or the Boston–Mathias extrapolation of the alpha function. The RK-ASPEN model is indicated by ASPEN PLUS user guide to this application due to the before mention characteristic, as well as the formulation of the mixing rules. This model was validated by [25]. For the simulation CO_2_ was considered and Henry component. The thermodynamic model used to represent the low pressure processes was UNIQUAC model. This model correctly represents ethanol-water systems and was validated by [26]. Although the methods mentioned were the best available, it is recognized that for complex streams as present in our process the model will never be 100% accurate.

The flowcharts of the Aspen Plus^®^ process simulation is presented in Figure A1, Figure A2, Figure A3 and Figure A4.

Table A1 summarizes all the simulated equipment and the specification set in the Aspen Plus^®^ simulator. Note that Aspen Plus^®^ does not have a specific model for representing solid-liquid extraction process with stationary bed of solid, but this does not limit the use of the software to evaluate mass and energy balance of this unit operation. The extraction process was simulated in ASPEN Plus using the ASPEN models mixer, heat exchanger and separator. In the first model solvent and biomass was mixed at the desired pressure, than it was heated to extraction temperature and in the separator model the experimental SFE results were inferred by a design specification calculation tool (flowsheeting options, Design Specs). Thermodynamic equilibrium was calculated in the flash tank in which CO_2_ is separated. In this way, simulation could represent the process successfully.

**Table A1:** Equipment specification simulated in Aspen Plus^®^.

Equipment	Description	ASPEN Model	Parameters
*SFE*			
H-101	Solvent CO_2_ cooling	Exchangers-Heater	293 K
H-1101	Solvent CO_2_ cooling	Exchangers-Heater	263 K
P-101	Solvent CO_2_ pumping	Pressure Changers-Pump	20 MPa
H-102	Solvent CO_2_ heating	Exchangers-Heater	313 K
M-101	SFE extractor	Mixers-Mixer	Set according to the experimental data
H-104		Exchangers-Heater	
EX-101		Separators-Sep	
S-101	CO_2_ separation tank	Separators-flash2	5 Mpa; 298 K
*Fine particles separations*			
MILL	Mill	Mixers-Mixer	Pressure (set pressure constant)
S-301	Sieve	Separators-Sep	Set using the design specification to separate fine fraction with the chemical composition described in Alcázar-Alay [7]
*LPSE*			
H-202	Solvent ethanol heating	Exchangers-Heater	Set according to the experimental data
M-201	LPSE extractor	Mixers-Mixer	Set according to the experimental data
H-201		Exchangers-Heater	
S-201	Centrifuge	Separators-Sep	Set to separate the waste solid biomass from the extract and solvent
P-102	Ethanol evaporator	Pressure Changers-Valve	0.016 MPa
H-203		Exchangers-Heater	348 K
HEVA2		Separators-flash2	0.1 MPa
H-204	Solvent ethanol cooling	Exchangers-Heater	303 K
H-205	Product cooling	Exchangers-Heater	303 K

## Appendix B

OSMOSE platform allows one to link several software such as Belsim Vali and Aspen Plus^®^ for a complete suite of computation and result analysis tools (optimization, sensitivity analysis, Pareto curve analysis, *etc.*). In the present study energy and material flow modeling was done in Aspen Plus^®^ as described previously and the thermal integration modeling and the economic modeling was performed in the OSMOSE platform (Figure B1). OSMOSE simulation details are described in this appendix. Figure B1 illustrates the involved modeling methodology when OSMOSE was used, where information is exchanged across the models using state and optimized variables based on the decision variables or performance indicators. Note that only few tools of the OSMOSE platform was used in this study.

Based on the pinch analysis methodology [11], the optimal thermal process integration is computed in the OSMOSE platform after the maximum heat recovery potential between hot and cold streams is defined and a minimum approach temperature ΔT_min_, which represents the energy capital trade-off between the energy savings obtained by heat exchange and the required heat exchangers investment, is considered. The optimal utility integration is obtained when the combined production of fuel, power and heat are maximized, which minimizes the operating cost by solving a linear programming problem (*i.e.*, mathematical method used in computer modeling to find the best possible solution representing the problem using linear relationships). In the thermal integration, the primary energy requirement must be satisfied in terms of hot and cold utilities. The minimum energy requirement is computed from the hot and cold process streams using the heat cascade method, which accounts for the potential heat recovery. The potential fuels are assembled in a superstructure, which integrates different possibilities and computes the optimal solution by minimizing the operating cost using a linear programming model.

The thermal integration is declared in OSMOSE as follow. First the variables are extracted from Aspen Plus^®^ simulation (as in the tag assignment example), then the thermal integration is performed (thermal integration modeling).

Tag assignment example in MATLAB

% SFE extraction

% H-102

nt=nt+1;

technology.Tags(nt).TagName = {‘t_c2_in’};

technology.Tags(nt).Unit = {‘C’};

technology.Tags(nt).Aspen.Line_1 = {‘Stream-Var Stream=SFE.C2 Substream=MIXED’};

technology.Tags(nt).Aspen.Line_2 = {‘Variable=TEMP’};

technology.Tags(nt).Status = {‘off’};

Thermal integration modeling

% description of the flow

% type, unit, tag_name , T_in [K], h_in [kW], T_out [K], h_out[kW], deltaTmin

ns = 0;

% Cold streams () --------------------------------------------------------- all in kW

ns = ns+1;

 technology.EI.Streams(ns).Short         = {‘qt’,‘sfe’,‘h102’,‘@t_c2_in+273’,‘0’,‘@t_c2_out+273’,‘@heat_h102*1163’,5};

ns = ns+1;

 technology.EI.Streams(ns).Short         = {‘qt’,‘lpse’,‘h202’,‘@t_e2_in+273’,‘0’,‘@t_e2_out+273’,‘@heat_h202*1163’,5};

ns = ns+1;

 technology.EI.Streams(ns).Short         = {‘qt’,‘lpse’,‘h203’,‘@t_c203_in+273’,‘0’,‘@t_c203_out+273’,‘@heat_h203*1163’,5};

% Hot streams () --------------------------------------------------------------

ns = ns+1;

 technology.EI.Streams(ns).Short     =     {‘qt’,‘sfe’,‘h101’,‘@t_co2+273.15’,‘-1*@heat_h101*1163’,‘@t_c1+273.15’,‘0’,5};

ns = ns+1;

 technology.EI.Streams(ns).Short     =     {‘qt’,‘lpse’,‘h204’,‘@t_h204_in+273’,‘-1*@heat_h204*1163’,‘@t_h204_out+273’,‘0’,5};

ns = ns+1;

technology.EI.Streams(ns).Short     =     {‘qt’,‘lpse’,‘h205’,‘@t_h205_in+273.15’,‘-1*@heat_h205*1163’,‘@t_h205_out+273.15’,‘0’,5};

Using the data from the Aspen Plus^®^ and thermal process integration models, the costs are estimated in OSMOSE based on the equipment sizing and cost correlations from the literature [14,15]. Table B1 gives the cost function and the necessary parameter for its calculation for the evaluated equipment.

**Table B1:** Cost function for the evaluated equipment defined in the economic analysis simulated in OSMOSE.

Equipment	Cost Function	Reference
*SFE*		
SFE extractor	Jacketed reactor	[15]
CO_2_ separation tank	Flash drum 2 min	[14,15]
*Fine particles separations*		
Mill	Vibrating Ball Mill	[15]
Sieve	Vertical Vessels and Sieve Trays	[14,15]
*LPSE*		
LPSE extractor	Jacketed reactor	[15]
Centrifuge	Sedimentation Centrifuge	[15]
*HEN*	Heat exchange network	Calculated by OSMOSE after thermal integration

The electricity consumption modeling developed in OSMOSE is presented in the following.

%% Electricity consumption (kW)

%---------------------------------------------------------------------

% SFE process

power_sfe = power_pco2;

% LPSE process

% Stirring in the extraction reactor 0.3 kWh/m3

power_extraction = volum_lpse201 * 0.3;

% Separation centrifuge 4000 kW/(m3/s)

power_cent = volum_lpses201 * 4000/3600;

power_lpse = power_extraction + power_cent;

% MILLING

power_mill = 40 * massflow/size ^ 0.3;

power_sieve = 0.75; % reference manufactor

power_milling = power_mill + power_sieve;

%---------------------------------------------------------------------

%%% total electricity consumption

elec_consum = (power_sfe + power_lpse + power_milling) * 1.3; %excess of 30% is assumed
